# Keystroke Dynamics based Hybrid Nanogenerators for Biometric Authentication and Identification using Artificial Intelligence

**DOI:** 10.1002/advs.202100711

**Published:** 2021-06-02

**Authors:** Pukar Maharjan, Kumar Shrestha, Trilochan Bhatta, Hyunok Cho, Chani Park, Md Salauddin, M. Toyabur Rahman, SM Sohel Rana, Sanghyun Lee, Jae Y. Park

**Affiliations:** ^1^ Advanced Sensor and Energy Research Laboratory Department of Electronic Engineering Kwangwoon University Seoul 01897 Republic of Korea

**Keywords:** artificial intelligence, biometric authentication, keystroke dynamics, self‐powered sensors, triboelectric

## Abstract

Cyberattack is one of the severe threats in the digital world as it encompasses everything related to personal information, health, finances, intellectual properties, and even national security. Password‐based authentication is the most practiced authentication system, however, is vulnerable to several attacks such as dictionary attack, shoulder surfing attack, and guessing attack. Here, a new keystroke dynamics‐based hybrid nanogenerator for biometric authentication and identification integrated with artificial intelligence (AI) is reported. Keystroke dynamics offer behavioral and contextual information that can distinguish and authorize the individuals based on their typing rhythms. The hybrid electromagnetic‐triboelectric nanogenerators/sensors efficiently convert the keystroke mechanical energy into electrical signals, which are fed into an artificial neural network based AI system. The self‐powered hybrid sensors‐based biometric authentication system integrated with a neural network achieves an accuracy of 99% and offers a promising hybrid security layer against password vulnerability.

## Graphical abstract

Cybersecurity is one of the severe issues in this modern computing world where password‐based security is being more vulnerable. Herein, a keystroke dynamics‐based hybrid electromagnetic‐triboelectric nanogenerator for biometric authentication and identification system integrated with artificial intelligence is developed with an excellent accuracy of 99%.

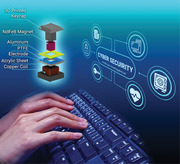

## 1 Introduction

Password‐based authentication system has been the most popular method for authenticating users in computing devices and system access, where keyboards are used to input PINs, tokens, or passwords. A keyboard is being commonly used as an input and control device for many purposes such as computing, security access, data recording, communication, financial, and so on. However, the password‐based authentication system is more fragile for weak passwords and prone to numerous attacks such as dictionary attacks, guessing attacks, and shoulder surfing attacks.^[^ ^1^ ^]^ When the password is exposed or acquired by a third‐party, it leads to a security breach which is extremely dangerous as confidential information regarding personal, security, financial, safety, and health will be exposed to unknown parties. To strengthen cybersecurity, two‐factor authentication (2FA), as well as multiple‐factor authentication, came forward. However, 2FA has also vulnerabilities that can be used by cybercriminals to bypass its security layer and apply different attacks like malware, phishing, man‐in‐the‐middle, or even account recovery schemes to avoid the 2FA features. Of late, keystroke dynamics and their use in enhancing user authentication is being hot topics of research due to the increasing necessity of high cybersecurity.^[^ ^2^, ^3^, ^4^ ^]^ Keystroke dynamics acquire behavioral information such as keystroke force, typing speed, latency between successive keystrokes, and hold time, of an individual which can be used to build a distinctive signature to be used for user identification and authentication.^[^ ^5^, ^6^, ^7^, ^8^ ^]^ It possesses user typing patterns and rhythms which are unique because of the neurophysiological mechanisms, similar to handwriting and signatures and contains sufficient information to serve as a potential biometric identifier. In comparison to other behavioral biometrics such as voice detection, handwriting, gait motion, and gestures, keystroke dynamics‐based biometric is non‐intrusive, convenient, cost‐effective, and capable of continuous authentication.^[^ ^9^ ^]^ Although the keystroke dynamics depend on external factors such as keyboard size, layout, and mechanisms, it is found that the frequently typed words and strings show more consistency.^[^ ^4^ ^]^ The detailed study of digraphs (time latencies between two consecutive keystrokes), trigraphs (time latencies between three consecutive keystrokes), and even *n*‐graphs make the keystroke biometric more secure and advanced. Moreover, the external hardware keyloggers which are supposed to be used for the study of a user interface, are found misused for intrusive accesses and cyber‐theft.

As a result, further research on keystroke dynamics‐based authentication with external sensors such as pressure sensors^[^ ^8^ ^]^ or even triboelectric nanogenerators (TENG)^[^ ^5^, ^6^, ^7^ ^]^ has been trending on. Through energy harvesting mechanisms such as triboelectrification, electromagnetism, and piezoelectricity, the sensor can be driven as a battery‐free sensing device since the sensor itself can produce an electrical signal upon external mechanical pressure and force.^[^ ^10^, ^11^, ^12^, ^13^, ^14^, ^15^, ^16^ ^]^ In the case of an electromagnetic generator (EMG), it can provide high current and low voltage while triboelectric can provide high voltage and low current. Especially under the low frequency‐based mechanical motion, TENG has superiority for generating high voltage which is difficult for EMG. However, EMG generates a large current under mechanical motion with high frequency. Therefore, hybridizing TENG‐EMG has been a great choice of research regarding the development of hybrid energy harvesters for various applications. Although the hybridization can be done with a piezoelectric and thermoelectric generator, the voltage and current output levels in these energy harvesting technologies are very low than the output from EMG and TENG. Besides energy harvesting property, many applications have been demonstrated by using triboelectric self‐powered sensors such as gesture detection,^[^ ^11^, ^17^ ^]^ pressure sensor,^[^ ^18^, ^19^, ^20^ ^]^ motion sensing,^[^ ^16^, ^21^, ^22^, ^23^ ^]^ etc. These external sensors help to collect more information regarding the behavioral features of user keystrokes for making the authentication more reliable. The individual sensor acts as a single layer of security in authentication while implementing a hybrid sensor system provides more layers of security resulting highly secure and accurate authentication system. This hybrid combination can operate under very low to high typing speed and typing force, which makes it more advantageous compared to a single individual sensing mechanism. Also, the EMG sensor can detect high typing speed as the output EMG voltage is proportional to the typing speed, while the TENG sensor can detect high typing force as the output TENG voltage is proportional to the typing force. The typing speed increases the change in magnetic flux while typing force increase the effective contact area between the two triboelectric layers. Moreover, by integrating artificial intelligence (AI) with these sensors, the system can efficiently learn, adapt, predict the sensor performance along with individual's behavioral information which can make the whole system more accurate, reliable, and sustainable.^[^ ^14^, ^24^, ^25^, ^26^, ^27^ ^]^

Taking a step ahead, in this work we propose a new keystroke biometric authentication based on hybrid electromagnetic‐triboelectric self‐powered sensors assisted by an artificial neural network (ANN). The self‐powered hybrid sensors are capable of continuously collect user's keystroke information in a non‐intrusive method. The keystroke mechanical energy is converted into electrical signals^[^ ^28^, ^29^, ^30^ ^]^ by the hybrid sensors and is processed in a custom signal processing unit for extracting behavioral features of the user which are fed into an ANN for user identification and authentication. Each sensor delivers keystroke behavioral information for typing motion such as typing force, holding time, flight time, interval, and so on, and provides double security layers toward the user authentication. Comparing to other machine learning platforms, deep learning neural networks can continuously learn and adapt user's keystroke behaviors. Since a person's behavioral nature might change based on the mood, surroundings, age, and accessibility, the neural network learns and adapts in the system to provide a more sophisticated personal security system. This battery‐less keystroke sensing system along with a neural network‐based software system can precisely distinguish the user and validate throughout their typing rhythm with an accuracy of 99%. With the features such as self‐powered sensors, dual authentication systems, and deep learning neural networks, this work offers a promising approach toward a highly secure layer in the computing industry with a behavioral biometric authentication system.

## 2 Results and Discussion

**Figure 1:**
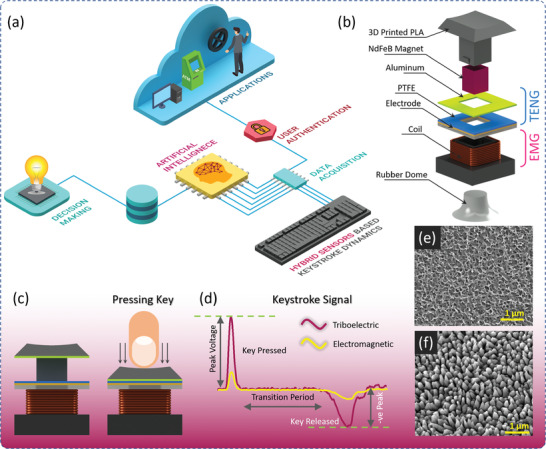
Self‐powered hybrid sensors driven biometric authentication system. a) Illustration of self‐powered hybrid sensors driven keystroke dynamics‐based biometric authentication system framework using AI. b) Schematic structure of the hybrid electromagnetic‐triboelectric sensor. c) Key pressing interaction of proposed hybrid sensor design. d) Keystroke output signal from the hybrid sensor under key pressed. e) FESEM image of surface‐modified Al film. f) FESEM image of surface‐modified PTFE film.

The proposed keystroke dynamics‐based behavioral biometric identification and authentication system using self‐powered hybrid sensors and AI is illustrated in **Figure** **1a**. The proposed hybrid sensors can be easily installed on commercial keyboards. The user's keystroke behavioral information is collected through hybrid sensors and a data acquisition system. The collected user data are forwarded to the AI model which extracts the user features and identifies users for authentication. The hybrid sensor is based on an EMG and TENG. The schematic structure of the hybridized electromagnetic‐triboelectric self‐powered keystroke sensor is shown in Figure 1b. The keycap and its leg were printed via a 3D printer. The EMG consists of a rectangular Neodymium magnet (5 × 5 × 5 mm, N35 grade) inside a 3D printed keycap leg and a rectangular copper coil (Ø 150 µm, 600 turns) placed around the keycap holder. This combination of keycap‐leg including a magnet and keycap holder with a coil establishes an electromagnetic energy harvester. The TENG is based on a single‐electrode contact‐separation mechanism in which Aluminum (Al) film is used as a positive triboelectric material and polytetrafluoroethylene (PTFE) film as a negative triboelectric material along with a copper electrode on the backside. The Al film was attached to the bottom of the keycap and the PTFE film was placed on the top of the coil. To optimize the triboelectric output performance, the nanostructures on PTFE and Al were optimized through multiple fabrication recipes. The nanograss‐like nanostructures on Al were obtained through the water‐assisted oxidation (WAO) method. For this, different WAO process time was applied, and tested the samples for output TENG current. Figure S1a–d, Supporting Information, shows the surface morphology of Al surface with different WAO process times, split into 20, 40, and 60 min. After 60 min of WAO process time, more dense and uniform nanograss structures were formed. Similarly, Figure S1e–h, Supporting Information, shows the surface morphology of inductively coupled plasma reactive ion etching (ICP‐RIE) of PTFE surface with different etching times 10, 20, and 30 s. After 30 s of etching, all the gold nanoparticles were etched away, and uniform nanopillar‐like nanostructures were formed on the surface. The fabrication details of the complete hybrid sensors are explained in the experimental methodologies section and the corresponding photographs of the prototype development are shown in Figure S2, Supporting Information.

Figure 1c illustrates the key pressing mechanism of the proposed hybrid keystroke sensor and the corresponding output keystroke electrical signal is shown in Figure 1d. The working principle of the EMG‐based keystroke sensor is illustrated in Figure S3, Supporting Information. The rubber dome which is available in the commercial keyboard act as the spring, and during a press and release both electromagnetic and triboelectric mechanism works simultaneously. During the key pressing motion, the magnet placed inside the key leg goes through the coil placed around the key holder, which induces an emf in the coil. For a single keypress and release, the EMG sensor outputs a complete cycle of electromagnetic signals. Figure S4, Supporting Information, illustrates the working principle of the TENG‐based keystroke sensor which is based on contact electrification and electrostatic induction. There are no charges on both triboelectric layers in the initial state. During the keypress motion, the Al film comes into contact with PTFE film, and electrons exchanged occurred according to their electron affinities. The Al film lost the electrons and became positively charged while the PTFE film gained electrons and became negatively charged. During the key release motion, charges transfer from Al to the PTFE surface through the external circuit that generated triboelectric current. On further keypress, the charges from PTFE transfer to Al layer through external circuit generating reverse current. Therefore, this contact‐separation cycle continues generating alternating triboelectric current during key press and release. Furthermore, to study the charge transfer mechanism and electric potential distribution between the triboelectric layers, a finite element analysis in COMSOL software was performed, as shown in Figure S5, Supporting Information. The simulation results help to understand the triboelectric voltage generation under different contact‐separation gap distances between both triboelectric layers. The simulation shows the increment in the triboelectric voltage with the increase in the gap distance, as shown in Figure S6, Supporting Information. Besides the gap distance, the contact surface area is another important parameter for improving the triboelectric performance. To enhance the triboelectric performance, surface modification of triboelectric materials was implemented which helps to increase the effective surface contact area as well as output triboelectric voltage, during contact and separation.^[^ ^31^ ^]^ The fabrication details of the surface modification on Al film and PTFE film is explained under the methods section. Figure 1e,f shows the field emission scanning electron microscope (FESEM) images of the surface‐modified Al and PTFE film with nanostructures on the surface which depicts the uniform distribution of nanostructures throughout the area. To optimize the TENG performance of the sensor, the nanostructures were optimized and confirmed through FESEM and electric output performance.

**Figure 2:**
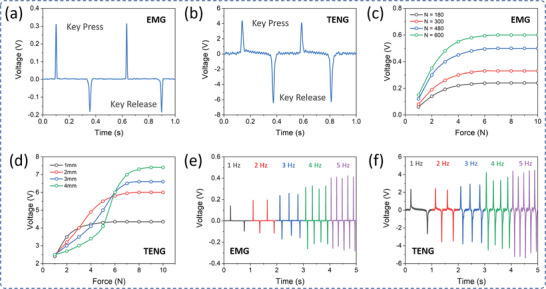
Electrical characterization of the hybrid sensor. a) Output open‐circuit voltage from EMG under key press and release. b) Output open‐circuit voltage from TENG under key press and release. c) Variation of EMG open‐circuit voltage under different keypress forces for different coil turns. d) Variation of TENG open‐circuit voltage under different keypress forces for the different number of gap distances between two triboelectric layers. e) Frequency response of EMG open‐circuit voltage. f) Frequency response of TENG open‐circuit voltage.

The sensor characteristics are essential for user authentication. To characterize the sensor performance under different pressure ranges and input force, a force gauge with a controlled pressure facility was used as an external pressure source. **Figure** **2a**,b is the output open‐circuit voltage waveforms of EMG and TENG sensor, respectively, under a keypress and release with a force of 5 N. The positive voltage peak indicates keypress while the negative voltage peak signifies key release. Similarly, the output short‐circuits current of EMG and TENG sensor is shown in Figure S7, Supporting Information. The EMG output voltage also depends on the number of coil turns. Depending on the available space in the commercial keyboard, the coil turn number was varied from 180 to 600 turns. Figure 2c shows the improvement in EMG output peak‐to‐peak voltage under different key pressing forces for a variety of coil turns and among them, a coil with 600 turns delivers the highest voltage. Since the output performance of the TENG sensor depends on the effective contact area, the nanostructures on the triboelectric surface need to be optimized. For this, various steps were considered during the fabrication of the nanostructures such as etching time, and thickness, as shown in Figure S1, Supporting Information. Depending upon the nanostructures, the TENG output performance was also compared and optimized as shown in Figure S8, Supporting Information. The Al surface with nanostructures developed via WAO for 60 min of processing time gives the best output current. In the case of PTFE film ICP‐RIE etching time, after 30 s etching, the output current was increased for more than 8 µA. In comparison to thickness, 50 µm of PTFE film performs better than thicker films. The quality of nanostructures on the PTFE surface depends on the gold nanoparticles as Au‐mask deposited through sputtering, therefore, a thicker Au‐mask requires a larger etching time and could damage the PTFE film itself. Here, 5 nm of Au‐mask shows the best results to create the nanopillar‐like nanostructures on the PTFE film. The triboelectric output voltage response for different gap distances between the two triboelectric layers is shown in Figure 2d, Supporting Information. When the input keypress force exceeds 6 N, the output voltage undergoes saturation. Since the keyboard typing speed varies from person to person, speed is an important parameter in keystroke study. Figure 2e,f shows the frequency response for EMG and TENG sensor output voltage, which gradually increase with the increment of keystroke frequency. The nanostructures in the triboelectric layers significantly increase the effective contact area under the increasing keypress force and frequency, which efficiently increases the triboelectric output voltage.^[^ ^31^ ^]^

**Figure 3:**
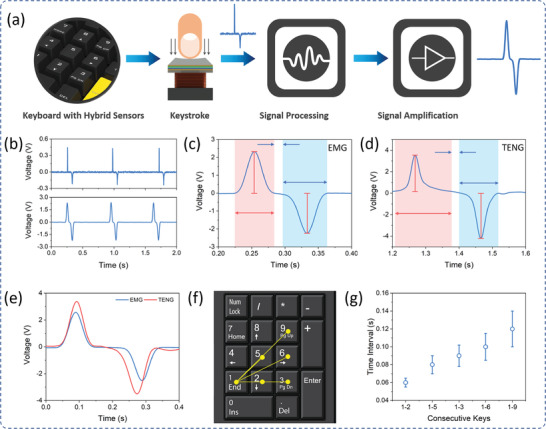
Data acquisition from hybrid sensors. a) Schematic illustration of a data acquisition system for converting keystroke signal into electrical data. b) Keystroke electrical signal before (top) and after (bottom) signal processing. c) Keystroke output voltage signal from EMG and the parameters for data acquisition. d) Keystroke output voltage signal from TENG and the parameters for data acquisition. e) Simultaneous EMG and TENG signal under a single keystroke. f) Illustration of distinct time intervals between different keys in a general numpad. g) Time interval between the different combinations of two consecutive keys.

**Figure 4:**
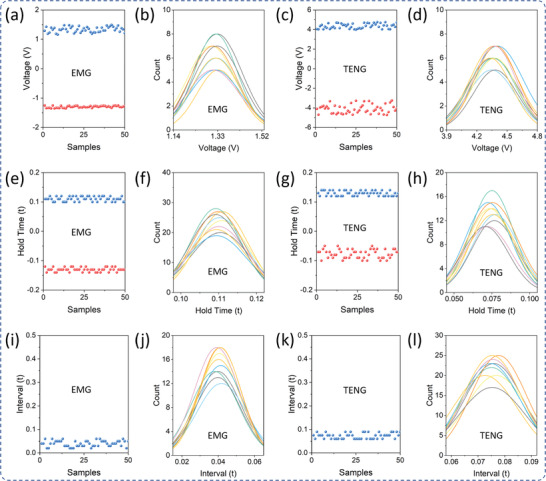
Keystroke dynamics features from EMG and TENG sensor multiple samples. a) Positive and negative peak voltages from the EMG sensor. b) Probability density functions of EMG positive voltage peaks for consecutive 50 keystrokes. c) Positive and negative peak voltages from TENG sensor. d) Probability density functions of TENG positive voltage peaks for consecutive 50 keystrokes. e) Hold times from positive and negative voltages of the EMG sensor. f) Probability density functions of hold times from EMG positive voltage peaks for consecutive 50 keystrokes. g) Hold times from positive and negative voltages of the TENG sensor. h) Probability density functions of hold times from TENG positive voltage peaks for consecutive 50 keystrokes. i) Time interval between two consecutive keystrokes from the EMG sensor. j) Probability density functions of the time interval between two consecutive keystrokes from the EMG sensor. k) Time interval between two consecutive keystrokes from the TENG sensor. l) Probability density functions of the time interval between two consecutive keystrokes from the TENG sensor.

To acquire the keystroke information for the biometric authentication, first, the raw sensor signals were processed through a customized multichannel signal processing and data acquisition system. **Figure** **3a** illustrates the keystroke signal acquisition and processing system consisting of low pass filters and amplifiers. The sensor raw output voltage signal consists of noise, which is filtered, and the low output voltage was amplified to prepare for the keystroke dataset. Figure 3b shows the EMG sensor signal before (top) and after (bottom) signal processing. To prepare the keystroke dataset for biometric authentication, different keystroke features such as signal amplitudes, hold time, interval, flight time, latencies, are extracted using data acquisition. These features were extracted from the electrical keystroke signals from both EMG and TENG sensors, as illustrated in Figure 3c,d, respectively. For each keystroke, the corresponding voltage signals from hybrid sensors have significant amplitude while other keys have negligible voltage levels. The acquired voltage signals from a hybrid sensor for a single keystroke are shown in Figure 3e. The TENG signal has originally amplitude of more than 3 V, while that of EMG was amplified using an amplifier. These voltage amplitudes hugely depend on the applied keystroke force. Besides force, the time interval between each key in the keyboard numpad depends on the distance between each key pairs, as shown in Figure 3f. This distance between two consecutive keys resembles the time interval between the corresponding keystroke signals. For a regular keyboard numpad, the distinct key pairs with distinct distances are 1–2, 1–5, 1–3, 1–6, and 1–9 and their corresponding time intervals acquired from multiple keystrokes are shown in Figure 3g. To build an AI model, numerous keystroke dynamic samples were observed along with their behavioral features. For biometric authentication, consistency in the keystroke dynamics is very important, however, it is behavioral information that might change on user's mental and physical conditions. Therefore, the AI model needs to learn, adapt, and implement the user's behavioral information and its effect on user identification and authentication. **Figure** **4a** shows the positive and negative amplitude from the EMG sensor collected from a single user for consecutive 50 samples. And its probability density function in Figure 4b shows the variance of the magnitude for a single user. Similarly, Figure 4c,d shows the 50 consecutive samples of both positive and negative amplitudes for the TENG sensor and its probability density function, respectively. From Figure 4e–h, it is observed that the TENG sensor has a comparatively consistent hold time during key pressing than key release. Since the TENG sensor signal has low width, the time interval between the keypress peak and release peak is larger compared to that of the EMG sensor, as shown in Figure 4i–l. In contrast, the EMG sensor produces more consistent keystroke features than TENG sensors, however, the hybrid sensor will provide additional comparative analysis which makes the system more advanced.

**Figure 5:**
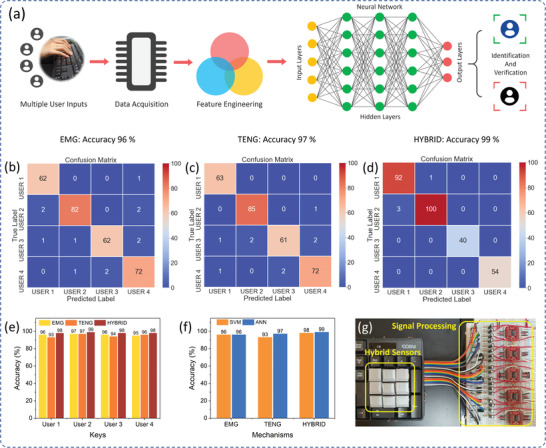
Self‐powered hybrid sensor‐driven keystroke dynamics‐based biometric authentication using a neural network. a) Procedure of proposed keystroke dynamics‐based user identification and authentication system using a neural network. Difference in accuracy scores for user authentication based on b) EMG sensors only, c) TENG sensors only, and d) hybrid EMG‐TENG sensors. e) Accuracy score matrices for different four users during user authentication. f) Comparison of accuracy score of user identification based on the machine learning model (SVM) and neural network model (ANN). g) Photograph of the fabricated sensors assembled keyboard with a signal processing unit.

For biometric authentication, the acquired voltage signals from the hybrid sensors are classified by the neural network, which can deeply analyze the user's keystroke behavioral data and effectively enhance the accuracy and robustness of the authentication system. An ANN based customized neural model was developed along with hybrid self‐powered sensors to build a biometric authentication system. In this multiclass ANN model, for every training set, we performed a forward pass using the weights and calculate the output of each node in the neural network. The last nodes lead to the final outputs which were then compared with the actual target in the training data to measure the errors using a loss function “Categorical Cross Entropy”. The loss function is a method of evaluating how well our ANN algorithm models the dataset. If the predictions are completely off, the loss function will output a higher number and vice versa. Since the algorithm needs to predict the multi‐class, an activation function “Softmax” was used which decides whether a neuron should be activated or not by calculating weighted sum and further adding bias with it. A gradient descent optimization algorithms “Adam” was implemented which minimizes the loss function by adjusting weights and bias. **Figure** **5a** illustrates a system architecture of a keystroke dynamics‐based biometric authentication system along with a flow diagram consisting of data acquisition and ANN‐based AI process. The hybrid sensors convert the keystroke mechanical energy into electrical signals, which undergoes signal processing and filtering process. A customized data acquisition system extracts the keystroke features such as signal magnitude, hold time, interval, and flight time from the sensor signals. In the experiment, four users were alternatively asked to enter a common password “1356” for 500 times. Each sample collected from the hybrid sensor has a total of 44 keystroke features consisting of 22 features from the EMG sensor and 22 features from the TENG sensor. Among 500 keystroke samples from each user, around 400 random samples were used as a training set for user identification via supervised learning while remaining as a test set for user identification/authentication. To study the benefit of hybrid sensors, the neural model was trained separately with EMG sensors data only, TENG sensors data only, and finally with hybrid sensor data, and the corresponding model accuracy during training is shown in Figure S9, Supporting Information. And the figures depict that the hybrid sensor exhibits higher accuracy.

Before user authentication, we performed user identification based on individual keystroke dynamics. For the four‐digit password, the keystroke features of those four keys were sent to the ANN model to identify the user's individual behavioral information. For each user (User 1, User 2, User 3, User 4), we performed the user identification with the EMG sensor, TENG sensor, and hybrid sensor data, as shown in Figures S10 and S11, Supporting Information. The confusion matrices show that, during user identification, our ANN algorithm achieves an accuracy of 98–99% for hybrid sensors, while it varies from 93–97% for a single EMG or TENG sensor. Then we trained our AI model with multiple user keystroke information for the common password “1356” for user authentication. Figure 5b–d shows the confusion matrix for user authentication based on the EMG sensor, TENG sensor, and hybrid sensor, respectively. While testing with “User 2” data, with only EMG sensor, the system achieved an accuracy of 96%, with TENG 97%, and with hybrid, 99% accuracy was achieved. These results show a clear benefit of using a hybrid sensor for increased accuracy in biometric authentication. We also performed user authentication with different user dataset, and the result is shown in Figure 5e, in which hybrid sensor plays better than the single sensor‐based user authentication. These excellent results exhibit a promising addition of a security layer in computing where password leakage might be no more things to be concerned about. Most of the state‐of‐the‐art works used support vector based machine learning (SVM) instead of a deep neural network.^[^ ^5^, ^6^ ^]^ We compared the user authentication with the same dataset for EMG, TENG, and hybrid sensors, as shown in Figure 5f. The comparative analysis shows that, for EMG sensors, there is no significant difference between SVM and ANN, however, ANN performed better for TENG and hybrid sensors with more accuracy. Also, ANN has the capability to learn the user's time‐variant keystroke behavioral information and update the algorithm, which is not possible in machine learning. Therefore, an ANN‐based neural network is implemented in this work. Figure 5g shows a photograph of the fabricated hybrid sensors installed on a commercial keyboard and analog circuit for signal processing.

## 3 Conclusion

In summary, we developed a new self‐powered hybrid nanogenerator/sensor for a keystroke dynamics‐driven biometric authentication system integrated with neural network‐based AI. The electromagnetic‐triboelectric principle‐based self‐powered hybrid sensors can efficiently collect the user's behavioral information from keystroke dynamics during key typing. A customized data acquisition and signal processing system was developed to acquire keystroke information and processing. Our ANN‐based AI model for user identification and authentication achieves high accuracy of 99% in user authentication using hybrid sensors even though a common password among multiple users was used. This remarkable success shows a promising cybersecurity layer over a password vulnerability. Compared to individual sensor‐based authentication, this hybrid sensor offers high accuracy and double security, under the same key dimension. More importantly, the proposed hybrid sensor can be fabricated using a 3D printer and quickly install on the commercial keyboard. In general, this work reveals a new possibility of a high cybersecurity layer in the computation world where password leak might be of no concern by using battery‐free hybrid sensors for user identification and authentication.

## 4 Experimental Section

### Fabrication of Aluminum Nanostructures

A thin film (130 µm, 99.9%) of Aluminum was obtained from Merck, Korea. The thin‐film Al was cleaned step‐by‐step using acetone, ethanol, and DI water. Then the film was boiled for 1 h in DI water and then dried in a convection oven for 2 h at 85 °C. This WAO process created uniform nanograss like structures on Al film. Finally, the film was cut into a sample size of 18 mm × 18 mm with a square hole of 8 mm × 8 mm.

### Fabrication of PTFE Nanostructures

A thin film (50 µm) of PTFE was obtained from Alphaflon, Korea. The PTFE film was cleaned thoroughly using isopropyl alcohol and DI water. A thin layer of gold nanoparticles (5 nm) was deposited using DC sputter as a mask for creating uniformly distributed nanostructures. Then the film underwent ICP‐RIE. In the ICP chamber Ar, O2, and CF4 gases were supplied at a flow rate of 15, 10, and 30 sccm, respectively. The etching process was performed for 30 s where the PTFE surface was etched away between the Au nanoparticles, resulting in nano‐pillars like nanostructures on the surface.

### Fabrication of Hybrid Electromagnetic‐Triboelectric Keystroke Sensor

The keycap and keycap leg was designed in CAD software (AutoCAD) and printed using a 3D printer (Ultimaker 3) using polylactic acid material. The size and dimensions of the keycap and keycap leg were the same as the commercial keyboard size, except for a modification to place a small magnet inside the keycap leg. A small rectangular neodymium magnet (5 mm × 5 mm × 5 mm, N35 grade) was placed inside the keycap leg. A copper coil (150 µm wire diameter, 600 turns) was fabricated and placed around the keycap holder. This pair of the copper coil and neodymium magnet built an EMG. Then a thin Al film with nanostructures on its surface as a top triboelectric material was attached to the bottom of the keycap using a double side adhesive tape. To place bottom triboelectric material, first, a thin rectangular acrylic sheet (with a rectangular hole at the middle) was placed over the copper coil. Then a nanostructured PTFE film along with a copper electrode was placed on the acrylic sheet using double side adhesive tape. This pair of Al and PTFE film formed a single electrode‐based TENG. The rubber dome available in the commercial keyboard was used as a spring mechanism. When the keycap leg with magnet was pressed down, the rubber dome tended it back to top with additional spring force.

### Measurement and Characterization

The output voltage and current were measured using a digital oscilloscope (WaveSurfer 510, Teledyne Lecroy) and an electrometer (Keithley 6514), respectively. The simulation for an electric potential distribution in triboelectric and magnetic flux density simulation in electromagnetic was performed in COMSOL and FEMM software, respectively. The input force was measured using force gauge (JSV‐H1000, Japan Instrumental System Co. Ltd.) The surface morphology of PTFE and Al film was characterized using a FESEM (Steroscan 440, Leica Cambridge).

## Conflict of Interest

The authors declare no conflict of interest.

## Supporting information

Supporting Information

## Data Availability

Research data are not shared.
