# Supplementary material for: Keystroke Dynamics based Hybrid Nanogenerators for Biometric Authentication and Identification using Artificial Intelligence
Source: Adv Sci (Weinh). 2021 Jun 2;8(15):2100711. doi: 10.1002/advs.202100711 (PMC8336502; doi:10.1002/advs.202100711)
Supplement: Supplementary file 1 — Supporting Information [file ADVS-8-2100711-s001.pdf]

© 2021 Wiley-VCH GmbH

ADVANCED SCIENCE
Open Access

Supporting Information

for *Adv. Sci.*, DOI: 10.1002/advs.202100711

Keystroke Dynamics based Hybrid Nanogenerators for Biometric Authentication and Identification using Artificial Intelligence

*Pukar Maharjan, Kumar Shrestha, Trilochan Bhatta, Hyunok Cho, Chani Park, Md Salauddin, MT Rahman, SM Sohel Rana, Sanghyun Lee, and Jae Y. Park**

Supporting Information

Keystroke Dynamics based Hybrid Nanogenerators for Biometric Authentication and Identification using Artificial Intelligence

*Pukar Maharjan, Kumar Shrestha, Trilochan Bhatta, Hyunok Cho, Chani Park, Md Salauddin, MT Rahman, SM Sohel Rana, Sanghyun Lee, and Jae Y. Park**

Advanced Sensor and Energy Research Laboratory, Department of Electronic Engineering, Kwangwoon University, Seoul 01897, Republic of Korea

Corresponding Author: Jae Y. Park (jaepark@kw.ac.kr)

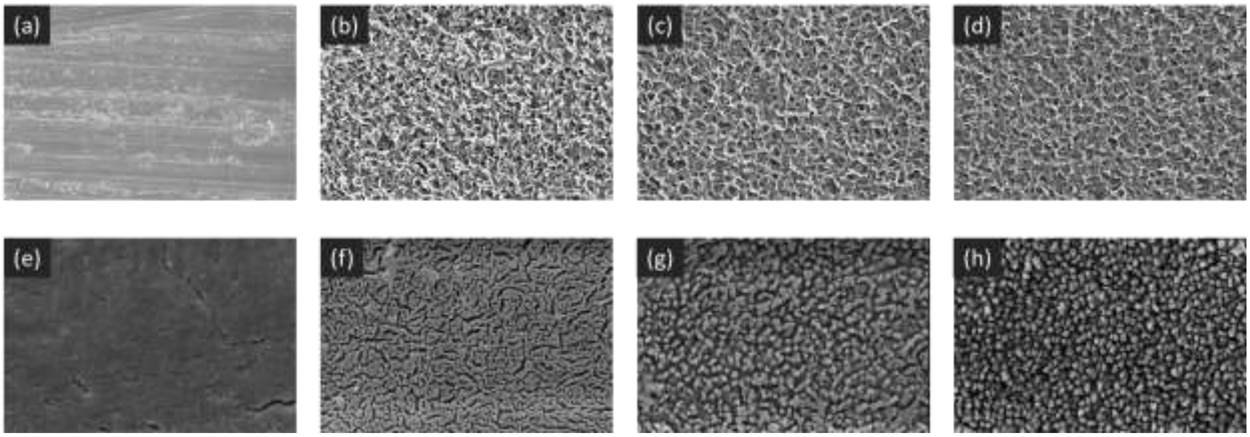

Figure S1. FESEM image of WAO Al surface with different WAO process time of (a) 0 min, (b) 20 min, (c) 40 min, and (d) 60 min. FESEM image of PTFE surface with different ICP-RIE etching time of (e) 0 s, (f) 10 s, (g) 20 s, and (h) 30 s.

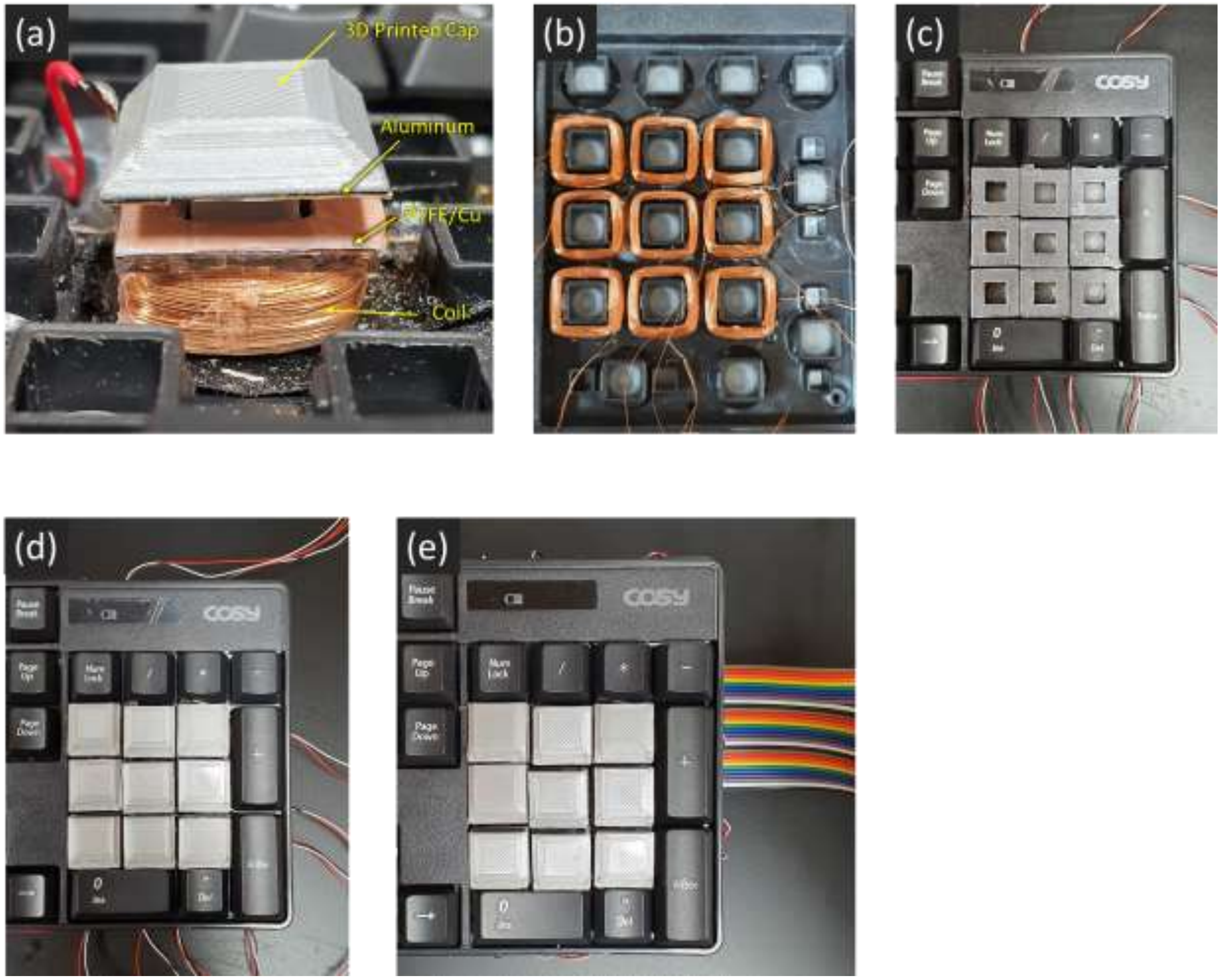

Figure S2. Fabrication of the self-powered hybrid sensors. (a) Photograph of the fabricated single hybrid sensor. (b) Photograph of installed multiple coils in numpad. (c) Photograph of installed TENG layers in numpad. (d) Photograph of installed 3d printed keycaps and final numpad. (e) Photograph of ready-to-use hybrid sensors installed numpad with a ribbon wire connector.

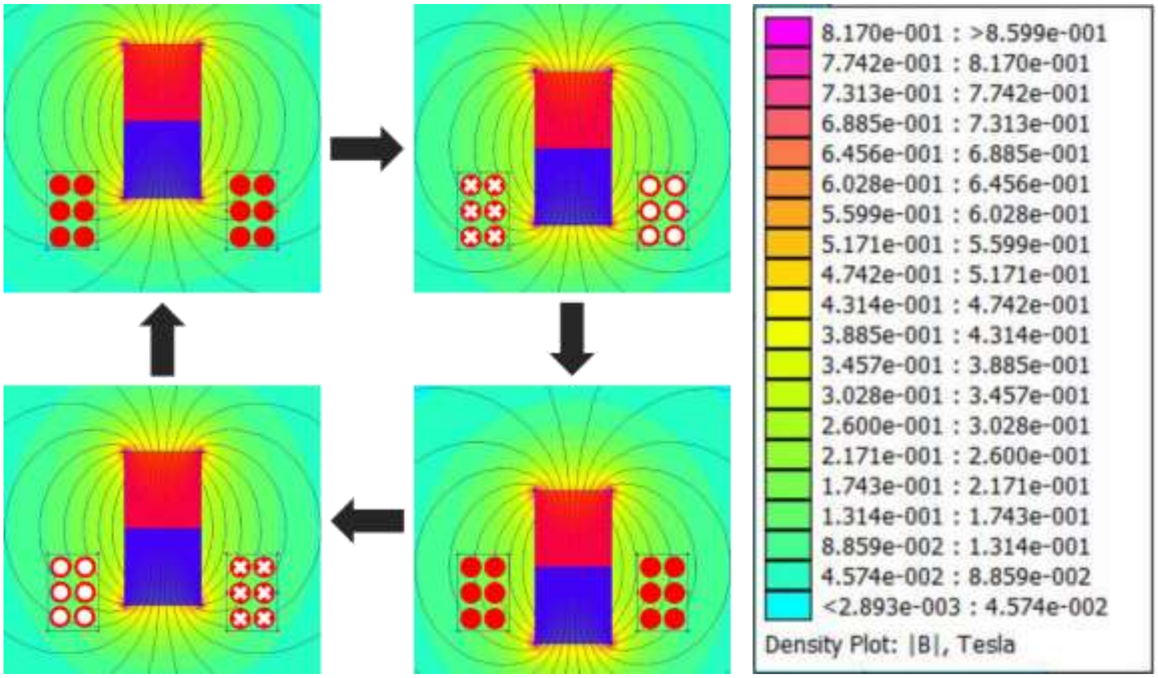

Figure S3. Working mechanism of EMG and FEMM simulation for distribution of magnetic flux density under keystroke activity.

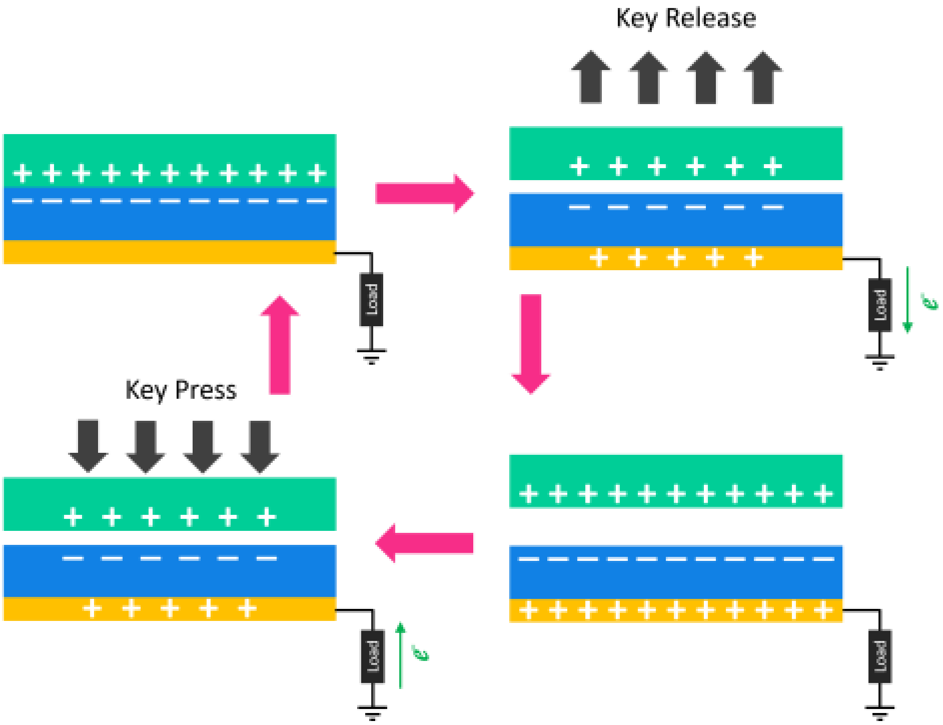

Figure S4. Working mechanism of the TENG based keystroke sensor.

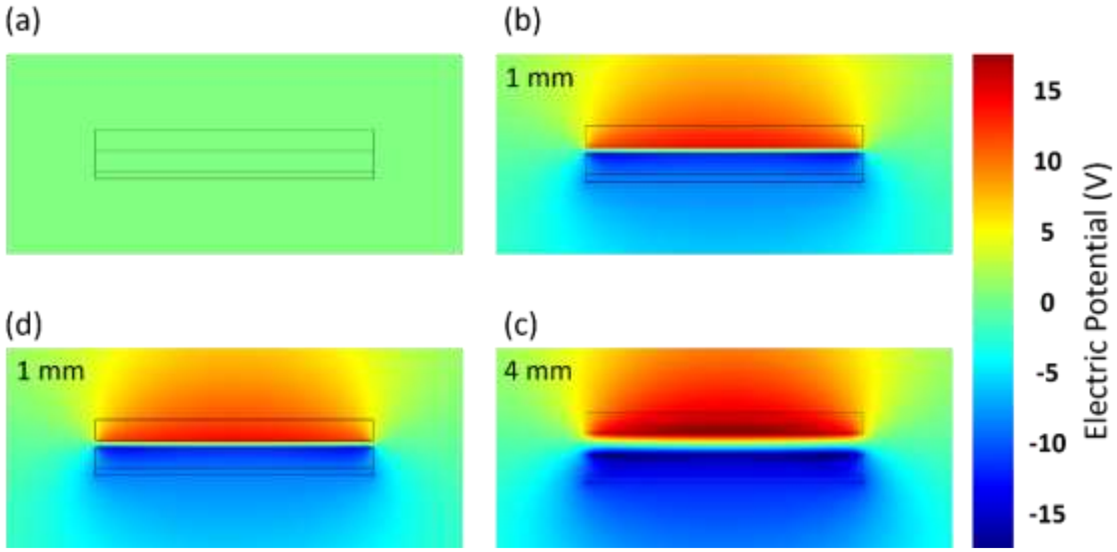

Figure S5. FEA simulation result of electric potential distribution in TENG performed in COMSOL.

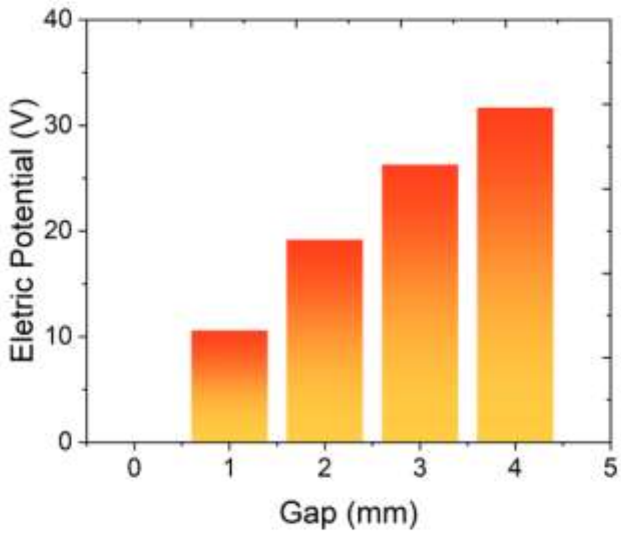

Figure S6. Simulated TENG electric potential under different gap distances between triboelectric layers.

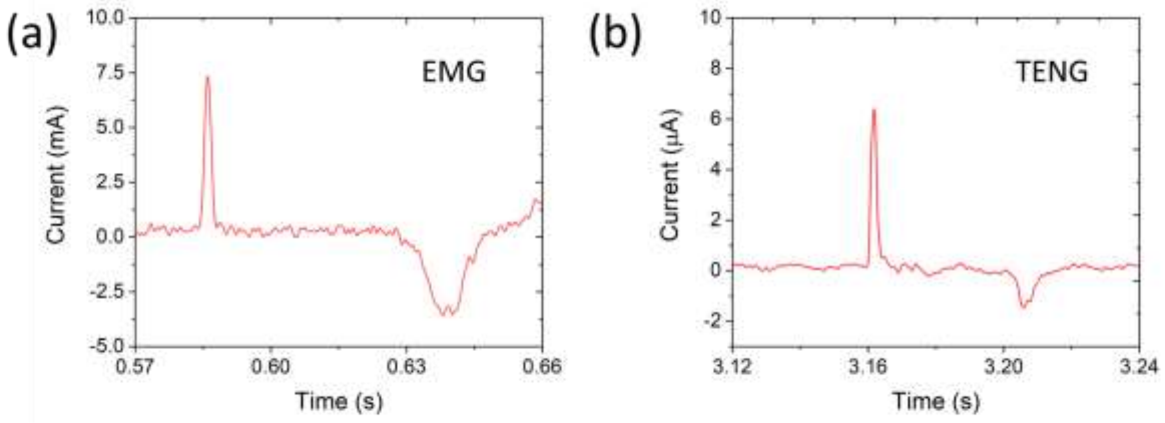

Figure S7. Output short-circuits current signals from (a) electromagnetic generator and (b) triboelectric nanogenerator.

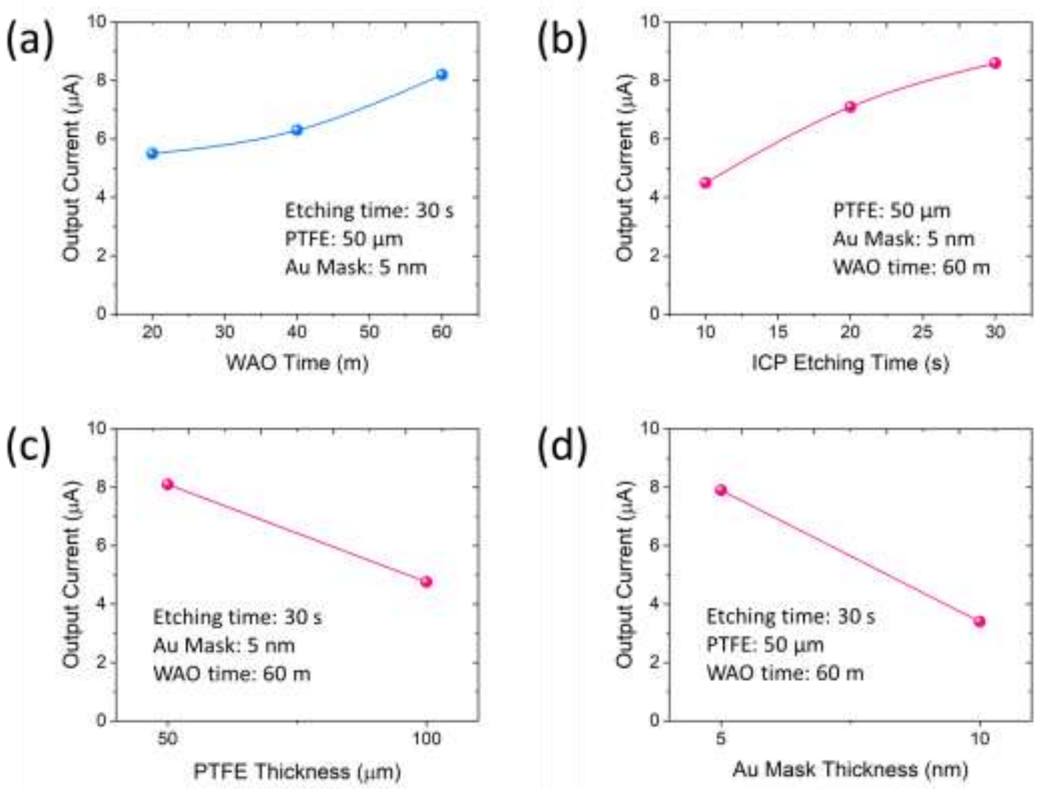

Figure S8. Optimization of nanostructures for TENG. (a) TENG Output current for nanostructured Al film paired with nanostructured PTFE film, under different WAO process time. (b) TENG Output current for nanostructured Al film paired with nanostructured PTFE film, under different PTFE ICP etching time. (c) TENG Output current for nanostructured Al film paired with nanostructured PTFE film, under different PTFE film thickness. (d) TENG Output current for nanostructured Al film paired with nanostructured PTFE film, under different Au mask thickness.

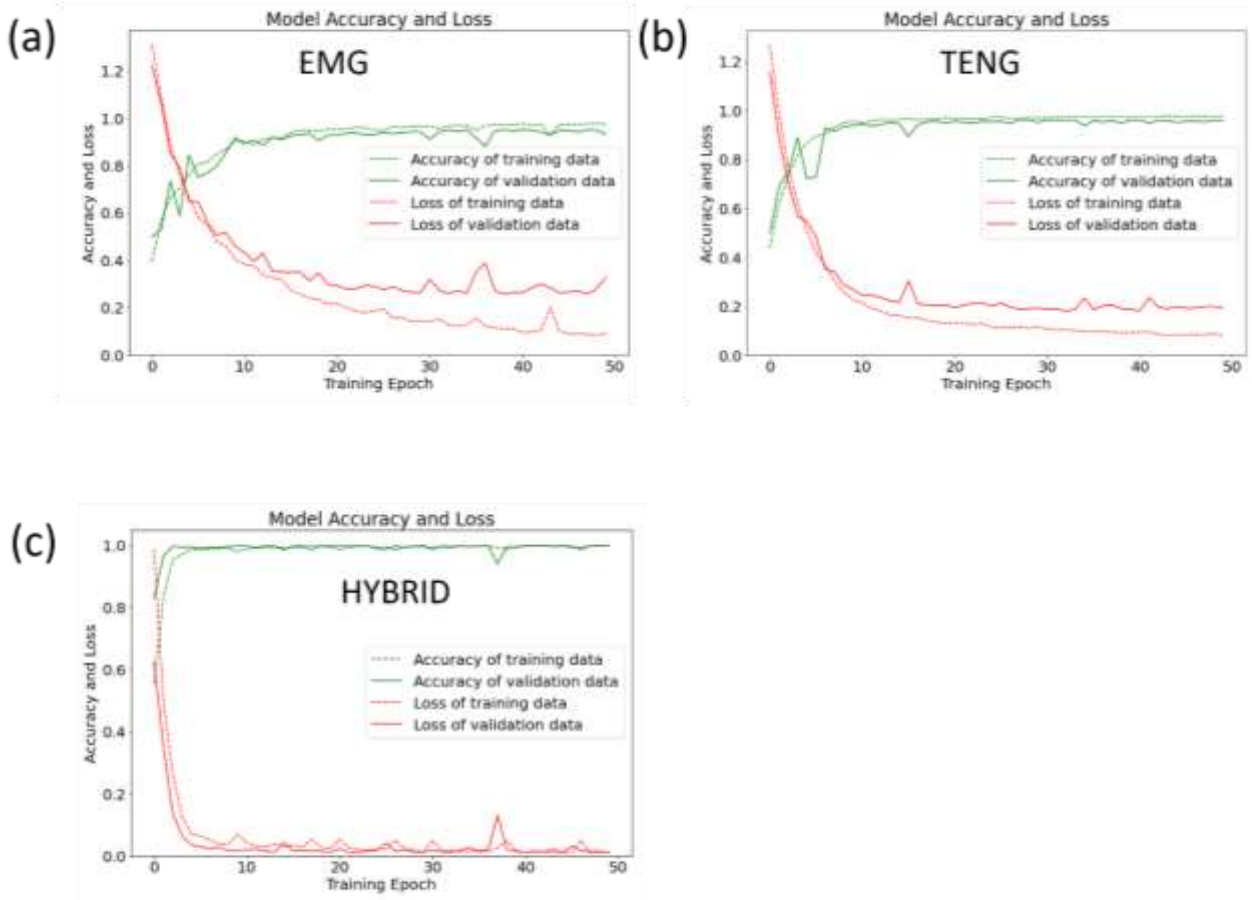

Figure S9. Artificial neural network model accuracy and loss during training with (a) EMG sensor data only, (b) TENG sensor data only, and (c) hybrid sensor data.

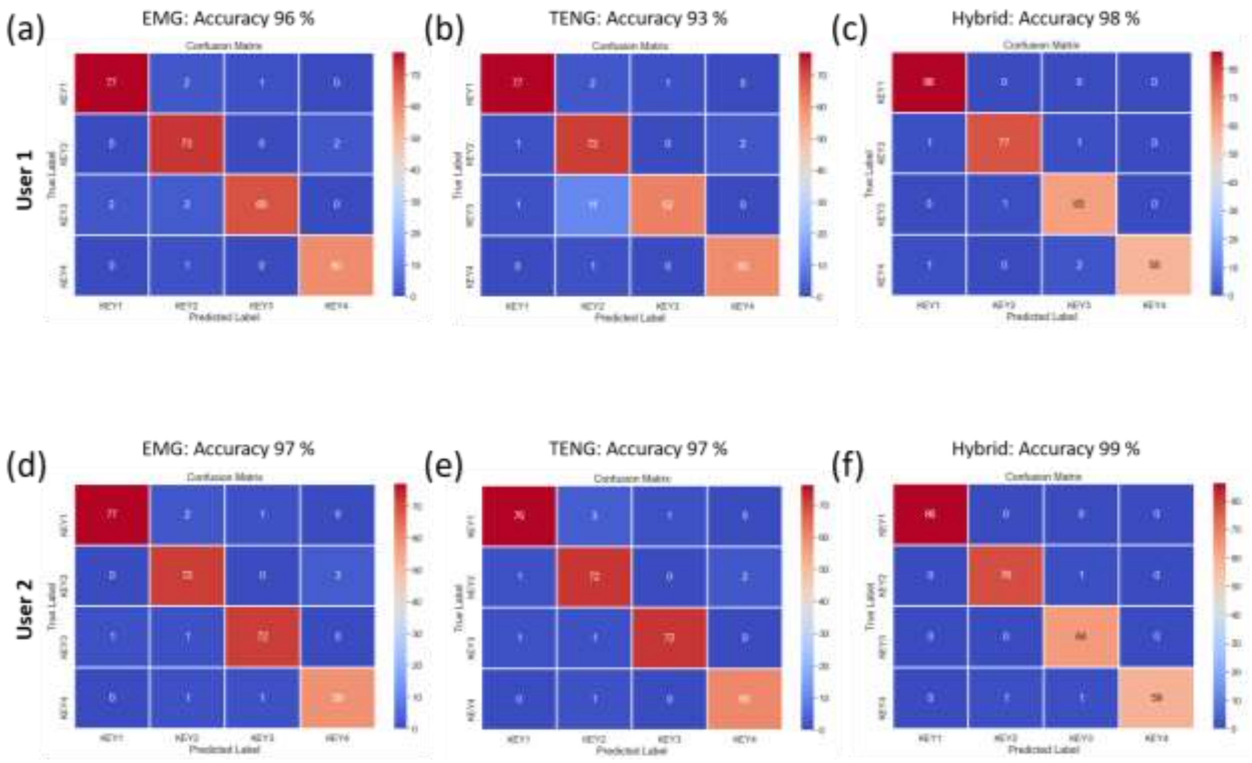

Figure S10. Keystroke dynamics-based user identification score matrices of user 1 by using (a) EMG sensors, (b) TENG sensors, and (c) hybrid sensors. Keystroke dynamics-based user identification score matrices of user 2 by using (d) EMG sensors, (e) TENG sensors, and (f) hybrid sensors.

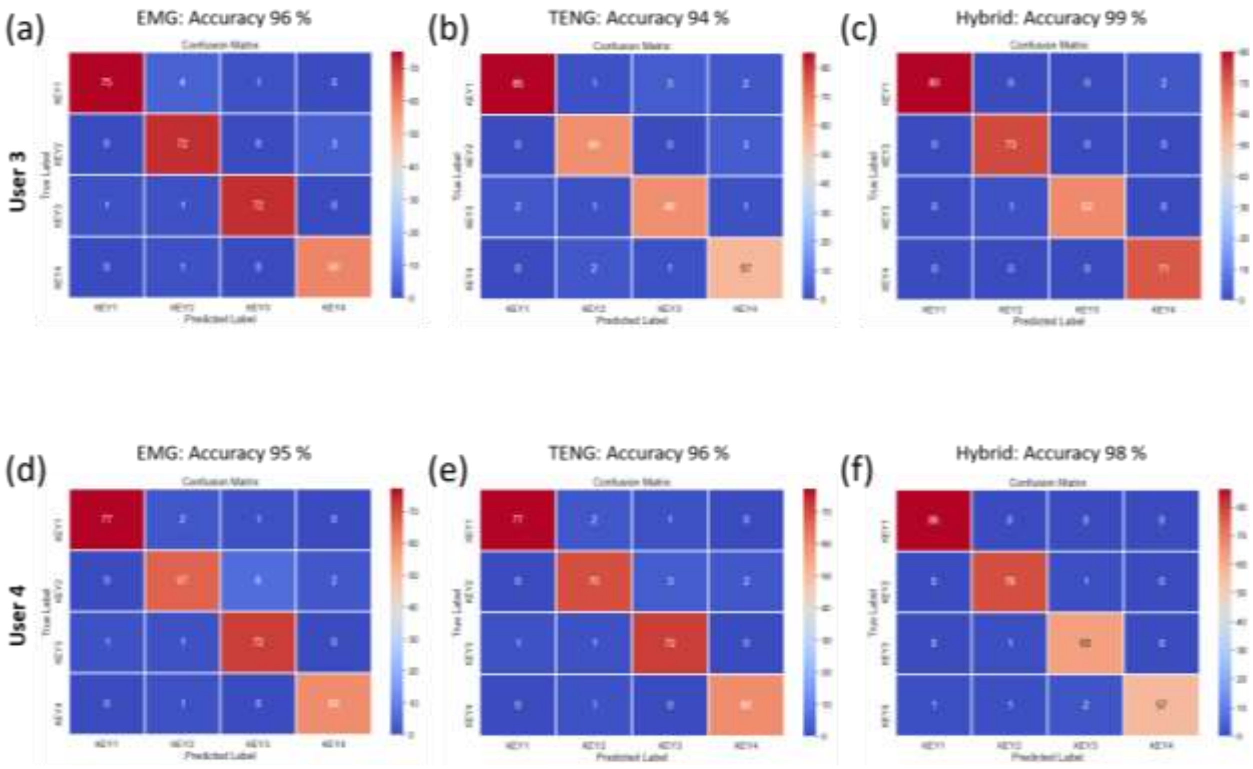

Figure S11. Keystroke dynamics-based user identification score matrices of user 3 by using (a) EMG sensors, (b) TENG sensors, and (c) hybrid sensors. Keystroke dynamics-based user identification score matrices of user 4 by using (d) EMG sensors, (e) TENG sensors, and (f) hybrid sensors.
